# Memantine/Aripiprazole Combination Alleviates Cognitive Dysfunction in Valproic Acid Rat Model of Autism: Hippocampal CREB/BDNF Signaling and Glutamate Homeostasis

**DOI:** 10.1007/s13311-023-01360-w

**Published:** 2023-03-14

**Authors:** Sohir M. Zohny, Mohamed Z. Habib, Magda I. Mohamad, Wael M. Elayat, Reham M. Elhossiny, Mohamed F. Abd El-Salam, Ghada A. M. Hassan, Sawsan Aboul-Fotouh

**Affiliations:** 1 Department of Clinical Pharmacology, Faculty of Medicine, Ain Shams University, Abbassia, Cairo, 11566 Egypt; 2 Department of Medical Biochemistry and Molecular Biology, Faculty of Medicine, Ain Shams University, Cairo, Egypt; 3 Department of Pediatrics, Faculty of Medicine, Ain Shams University, Cairo, Egypt; 4 Physiology Department, Faculty of Medicine, Ain Shams University, Cairo, Egypt; 5 Neuropsychiatry Department, Faculty of Medicine, Galala University, Al Galala, Egypt; 6 Neuropsychiatry Department, Faculty of Medicine, Ain Shams University, Cairo, Egypt; 7 Clinical Pharmacology Unit, Faculty of Medicine, Ain Shams University, Cairo, Egypt

**Keywords:** Autism spectrum disorder, Aripiprazole, Memantine, Cognitive dysfunction, Glutamate, Astrocyte

## Abstract

Significant efforts are increasingly directed towards identifying novel therapeutic targets for autism spectrum disorder (ASD) with a rising role of aberrant glutamatergic transmission in the pathogenesis of ASD-associated cellular and behavioral deficits. This study aimed at investigating the role of chronic memantine (20 mg/kg/day) and aripiprazole (3 mg/kg/day) combination therapy in the management of prenatal sodium valproate (VPA)-induced autistic-like/cognitive deficits in male Wistar rats. Pregnant female rats received a single intraperitoneal injection of VPA (600 mg/kg) to induce autistic-like behaviors in their offspring. Prenatal VPA induced autistic-like symptoms (decreased social interaction and the appearance of stereotyped behavior) with deficits in spatial learning (in Morris water maze) and cognitive flexibility (in the attentional set-shifting task) in addition to decreased hippocampal protein levels of phosphorylated cAMP response element-binding protein (p-CREB), brain-derived neurotrophic factor (BDNF), and gene expression of glutamate transporter-1 (*Glt-1*) with a decline in GABA/glutamate ratio (both measured by HPLC). These were accompanied by the appearance of numerous neurofibrillary tangles (NFTs) with enhanced apoptosis in hippocampal sections. Memantine/aripiprazole combination increased the protein levels of p-CREB, BDNF, and *Glt-1* gene expression with restoration of GABA/glutamate balance, attenuation of VPA-induced neurodegenerative changes and autistic-like symptoms, and improvement of cognitive performance. This study draws attention to the favorable cognitive effects of memantine/aripiprazole combination in autistic subjects which could be mediated via enhancing CREB/BDNF signaling with increased expression of astrocytic *Glt-1* and restoration of GABA/glutamate balance, leading to inhibition of hippocampal NFTs formation and neuronal apoptosis.

**Supplementary Information:**

The online version contains supplementary material available at 10.1007/s13311-023-01360-w.

## Introduction

Autism spectrum disorder (ASD) is a childhood neuro-behavioral disorder characterized by social, behavioral, intellectual, and cognitive deficits [1]. ASD typically presents with impairments in social interaction and repetitive stereotyped patterns of behavior [2]. Nearly, 1% of children have ASD [3] and it is above 4 times more common in males than in females [3, 4]. Many individuals with ASD also have other interfering symptoms, including irritability (aggression, self-injurious behavior, and severe tantrums). Behavioral therapy is often helpful in decreasing these behaviors; however, pharmacological management of some ASD-associated maladaptive behaviors and cognitive dysfunction is frequently needed adjunctively [5].

The pathophysiology of ASD is poorly understood which represents a substantial challenge to developing specific pharmacotherapies. Indeed, a heterogeneous genetic background and multiple environmental factors (such as maternal exposure to viral infections, toxins, pesticides, and drugs such as valproic acid during pregnancy) are thought to be involved in the etiology of ASD [6].

A substantial body of evidence highlights the fundamental role of disturbed glutamatergic neurotransmission in the pathophysiology of ASD. Glutamatergic neurotransmission plays important roles in cognition, learning, and memory [7]. Nevertheless, the sustained increase in synaptic glutamate with subsequent over-stimulation of the N-methyl-d-aspartate (NMDA) ionotropic glutamatergic receptors could activate a cascade of events leading ultimately to cellular death, a phenomenon termed “glutamate excitotoxicity” [8].

Astrocytes are involved in the uptake of glutamate from synaptic space by excitatory amino acid transporter (EAAT2) in humans and glutamate transporter-1 (*GLT-1*) in rodents which is responsible for more than 90% of total glutamate uptake [9]. Indeed, there has been an increasing interest in the role of *GLT-1* in regulating the dynamic process of synaptic transmission [7].

Interestingly, the upregulation of *GLT-1* could improve cognitive performance in rats [10]. Several studies have linked the dysregulated *Glt-1* expression with impaired synaptic glutamate clearance to the pathogenesis of ASD [11]. In mice, loss of *GLT-1* induced synaptic over-excitability with the appearance of pathological repetitive behaviors [12]. Deletion of fragile X mental retardation protein, which is associated with ASD pathogenesis, decreased *GLT-1* expression and glutamate re-uptake, resulting in abnormal neuronal hyperexcitability [13].

The active phosphorylated cAMP response element-binding protein (p-CREB) is a major activator of astrocytic *Glt-1* gene expression through binding to CRE binding site on the *Glt-1* promoter, highlighting the role of CREB-mediated transcription in preserving glutamate balance, maintaining synaptic plasticity and preventing excitotoxicity [14]. In addition to enhancing *Glt-1* expression, p-CREB promotes the transcription of many genes essential for neuronal survival as the brain-derived neurotrophic factor (BDNF) [15]. In turn, BDNF stimulates its receptor, tropomyosin receptor kinase B (TrkB), and activates ERK (extracellular signal-regulated kinase) with subsequent phosphorylation/activation of CREB which may represent a positive feedback loop [16].

The phosphorylated CREB has been shown to induce the transcription of the anti-apoptotic regulator, B-cell lymphoma 2 (Bcl-2) [17] and decrease the expression of the pro-apoptotic mediators, Bcl-2-associated X protein (BAX) and Caspase-3 [18, 19]. Moreover, BDNF reduces the levels of the hyperphosphorylated tau protein [20] which may represent a key player in the cognitive dysfunction in autistic subjects [21].

Since 2009, aripiprazole (ARI) was FDA-approved for the treatment of ASD-associated behavioral dysregulation in children and adolescents [22]. Aripiprazole exhibits effectiveness in improving cognitive abilities in autistic children with mild to moderate cognitive impairment [23]. Notably, aripiprazole has been shown to increase BDNF and p-CREB in HT22 neuronal cells which have been widely used as an in vitro model for elucidating the mechanism of oxidative stress-induced neurotoxicity [24].

Memantine (MEM) is a non-competitive NMDA receptor antagonist approved for the management of moderate to severe Alzheimer’s disease [25]. Memantine attenuated the behavioral changes and the increase in brain oxidative stress in prenatal valproic acid-exposed autistic rats and mice [26, 27]. Interestingly, memantine has been shown to ameliorate memory impairment via enhancing CREB/BDNF signaling in chronic unpredictable stress-exposed rats [28]. Data from pediatric studies suggested good tolerability and potential clinical benefits of memantine in children with ASD; memantine improved receptive and expressive language, verbal recognition memory, social interaction, irritability, stereotypic behavior, and hyperactivity in children with ASD [29–32]. Nevertheless, there is still a lack of clinical evidence for the efficacy of memantine in treating autistic-like behaviors in children and adolescents.

To the best of our knowledge, the effects of either memantine or aripiprazole on CREB/BDNF signaling have not been investigated in autistic subjects hitherto. Accordingly, this study could be the first to investigate the effects of memantine/aripiprazole combined administration on autistic-like behaviors and cognitive functions in the prenatal valproate rat model of autism and to explore their effects on CREB/BDNF signaling, astrocytic *Glt-1* expression, and glutamate balance which could represent key pathological events associated with ASD.

## Materials and Methods

### Animals

Thirty-eight adult Wistar rats (18 males and 20 females) weighing 200–250 g (8 weeks of age) were purchased from the animal facility of the Nile company for pharmaceutical and chemical industries (El-Sawah, Cairo, Egypt) and raised in the department of Clinical Pharmacology, Faculty of Medicine, Ain Shams University (Cairo, Egypt). Rats were housed in groups of 4–5 rats of the same sex in plexiglass cages with a mesh wire cover. Adult rats were allowed 1 week of acclimatization before starting experiments. Animals were maintained at a temperature (22 ± 2 °C), relative humidity (55 ± 5%), 12 h light/dark cycle at 5:00 am–5:00 pm, and good ventilation. Rat chow pellets (Meladco, El-Obour, Egypt) and water were provided ad libitum and the cages were cleaned daily throughout the experiment unless otherwise recommended by the study protocol. All efforts were made to minimize animal suffering as well as to reduce the number of animals used. The study was conducted in accordance with the EU Directive 2010/63/EU for animal experiments and was approved by the Research Ethics Committee of the Faculty of Medicine, Ain Shams University (FMASU-REC). FMASU-REC operates under Federal Wide Assurance. NO. FWA 000,017,585. (Approval number FAMSU MS 633/2021).

### Drugs and Chemicals

Sodium valproate (VPA) (SCI Pharmtech Inc, Taoyuan, Taiwan) was supplied as a white powder to be dissolved in 0.9% saline and injected intraperitoneally (i.p.) at a dose of 600 mg/kg [33, 34]. Memantine hydrochloride (Procos S.P.A, Cameri, Italy) was supplied as a white crystalline powder to be dissolved in 0.9% saline and injected at a dose of 20 mg/kg/day, i.p. [35]. Aripiprazole (Hetero Labs Limited, Hyderabad, India) was supplied as an off-white crystalline powder to be dissolved in Tween 80 and injected at a dose of 3 mg/kg/day, i.p. [36]. All drugs administered were freshly prepared directly before their use.

### Prenatal VPA Model of Autism

Adult males and females were allowed to mate overnight (with one male and one female in the same cage). After matting, female rats were examined vaginally every morning for detection of pregnancy which was confirmed by either, the presence of a vaginal plug or the presence of spermatozoa in vaginal smears. Once confirmed, this was designated as gestational day 0.5 (G 0.5). On G 12.5, pregnant rats were randomly assigned to receive either a single injection of sodium VPA or a single injection of an equal amount of 0.9% saline at the same time [33, 34, 37].

Pregnant rats were housed individually until pups were delivered. The total number of litters was 20 with 6–11 pups/litter. Mothers were allowed to raise their pups until weaned on post-natal day 23 (PND23), afterwards, only male pups were enrolled in the study. To reduce litter-to-litter variations, male pups were randomly allocated to the experimental groups. The same was utilized during the allocation to different behavioral tests. Randomization was done using GraphPad StatMate, Software Inc., Version 1.01i (1998), CA, USA. Male pups were housed in plexiglass cages (42 × 25 × 15 cm) in groups of 4–5 rats. Adult females were used only once for breeding and then were excluded from the experiment.

### Experimental Groups

Ninety male Wistar rat pups were randomly divided into five groups (18 pups in each) as follows:

- *Control group:* male offspring rats from saline-treated female rats. This group received daily i.p injections of the vehicle starting on PND 24 till the end of the study.
- *VPA/VEH-treated group:* male offspring rats from VPA-treated female rats. This group received daily i.p injections of the vehicle (VEH) for 38 days starting on PND 24.
- *VPA/MEM treated group:* male offspring rats from VPA-treated female rats. This group received daily injections of memantine for 38 days starting on PND 24.
- *VPA/ARI treated group:* male offspring rats from VPA-treated female rats. This group received daily injections of aripiprazole for 38 days starting on PND 24.
- *VPA/MEM/ARI treated group*: male offspring rats from VPA-treated female rats. This group received daily injections of memantine followed by aripiprazole for 38 days starting on PND 24.

N.B: For the control and the VPA/VEH-treated groups, 9 rats per group were injected with 0.9% saline and the other 9 rats were injected with Tween 80 (1%). The results of the 2 groups were statistically compared and revealed no difference between the 2 groups, so they were pooled into control and VPA/VEH-treated groups.

### Body Weight and Behavioral Assessments

**Fig. 1:**
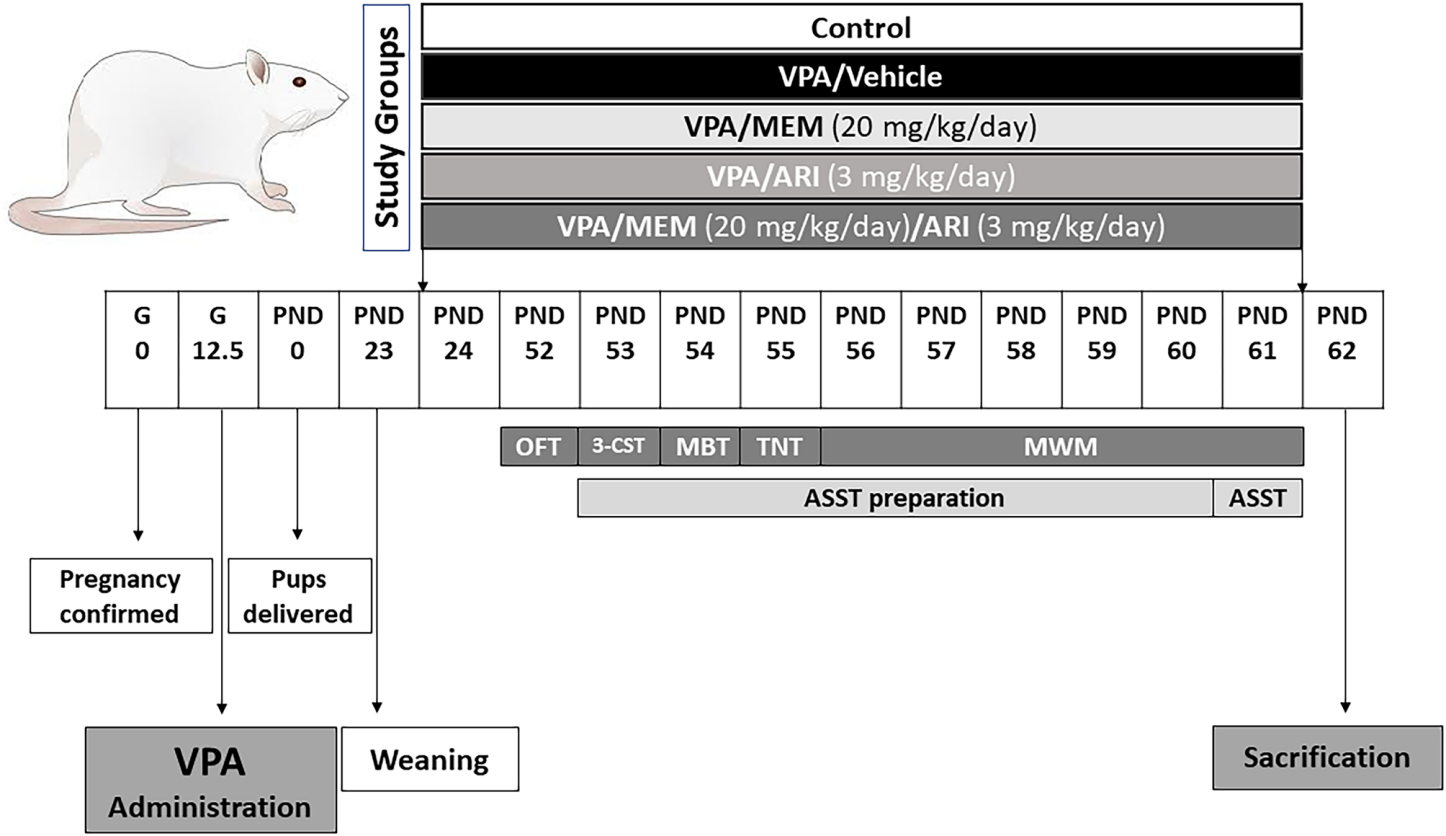
A diagram showing the experimental exposures of the study. *VPA* Sodium valproate, *MEM* Memantine, *ARI* Aripiprazole, *G* Gestational day, *PND* Postnatal day, *OFT* Open field test, *3-CST* Three-chamber sociability test, *MBT* Marble burying test, *TNT* Tail-immersion nociceptive test, *MWM* Morris water maze, *ASST* Attentional set-shifting task

Body weight was measured after weaning on PND23 and weekly thereafter. Eight rats per group were tested for the attentional set-shifting task and another ten rats per group were tested for the open field test, three-chamber sociability test, marble burying test, tail-immersion nociceptive test, and Morris water maze test. Rats’ behavior was videotaped, and a well-trained observer conducted the analysis manually from the previously recorded video. Observers were blinded to treatments during behavioral testing, histological and biochemical measurements. Each behavioral test was done on a separate day to reduce the influence of sequential testing. Treatments were received after the completion of the behavioral testing on the corresponding day. The timeline of the experimental protocol is presented in Fig. 1.

#### Open Field Test (OFT)

OFT was used to detect stereotyped behavior, general locomotor activity, and anxiety-related behaviors in rats. Rats were allowed to acclimatize to the test room 1 h before conducting the test. Each rat was placed individually in the center of a quadrangular apparatus (60 × 60 × 45 cm). The testing apparatus was divided into 16 equal squares and was illuminated by white light. Rat’s behavior was recorded over 5 min using a webcam. The following parameters were calculated: the total number of crossed squares (visited with all four feet), frequency of central zone entry, central zone duration, and frequency of grooming (face rubbing and licking or biting both paws and fur). The test was performed in a well-ventilated, darkened, and sound/light-attenuated testing room. The test arena was cleaned with 70% alcohol after each rat to remove any olfactory cues that may guide the animal’s behavior [38].

#### Three-Chamber Sociability Test (3-CST)

The 3-phase sociability test was performed as described by [34], using a three-chambered box (60 × 45 × 45 cm) with partitions dividing the box into three equal chambers with (10 × 10) rectangular openings allowing free exploration of the animals to the three chambers. During the habituation phase, the test rat was placed in the empty central chamber with the two lateral chambers containing two empty cylindrical wire cages (13.3 cm diameter × 21.0 cm height) and was allowed to freely explore all the three chambers for 5 min followed by the sociability phase, during which a novel rat (novel rat 1) with the same (strain, age, and sex) was placed into one of the lateral wire cages, while the other wire cage was left empty representing the (novel object). The test rat was left for 10 min, and times spent during exploration of the novel rat 1 or the novel object were used for calculation of the sociability index (SI). Afterward, the test rat went through the social novelty preference phase, during which a new unfamiliar rat was placed into the empty wire cage in the opposite chamber and was considered as (novel rat 2), while the other rat became now the (known rat). Again, the test rat was allowed 10 min to explore both sides, and times spent during exploration of the novel rat 2 or the known rat were used to calculate the social novelty preference index (SNI). Chambers were cleaned with 70% alcohol between tested animals.

The sociability index (SI) and the social novelty preference index (SNI) were calculated using the following equations:

$$\mathrm{SI}=\frac{(\mathrm{time\;exploring\;novel\;rat}\;1-\mathrm{time\;exploring\;novel\;object}) }{(\mathrm{time\;exploring\;novel\;rat}\;1 +\mathrm{ time\;exploring\;novel\;object})}$$

$$\mathrm{SNI}=\frac{(\mathrm{time\;exploring\;novel\;rat}\;2 -\mathrm{ time\;exploring\;known\;rat}) }{(\mathrm{time\;exploring \;novel\;rat}\;2 +\mathrm{ time\;exploring\;known\;rat})}$$

#### Marble Burying Test (MBT)

MBT was used to assess the repetitive stereotyped behavior, a core autistic symptom. During this test, a rat was placed individually in the center of a standard Plexiglas test cage with lighting conditions similar to the animal facility. Each test cage contained a 5 cm depth of clean bedding with 15 glass marbles (15 mm diameter) placed in each cage in evenly spaced 3 rows. The animal was tested for 20 min, then the number of buried marbles (at least 2/3 of it is covered with bedding) was counted [39].

#### Tail-Immersion Nociceptive Test (TNT)

TNT is a thermal test that evaluates sensitivity to a painful thermal stimulus. During this test, the lower 5 cm portion of the rat’s tail was immersed in a 55 °C maintained-temperature water bath. The latency for each rat to withdraw its tail from hot water was calculated by stopwatch, reflecting the nociceptive reaction time [34].

#### Morris Water Maze (MWM)

Morris water maze test was utilized to investigate hippocampus-dependent cognitive spatial learning and memory functions***.*** The test was conducted in a circular pool (1.8 m diameter) filled with water (25 °C ± 1) to a depth of 40 cm. A platform (10 cm diameter) located in the center of the northeast quadrant of the pool was submerged 1 cm under the water surface. Both pool and platform were made of polyvinyl plastic, black in color to prevent intra-maze cues which may guide escape behavior. Several constant extra maze visual cues (posters, objects, and equipment) were surrounding the maze so that the animal can learn to use the distal cues to navigate a direct path to the submerged platform when starting each trial from a different location. Each rat received 5 days of spatial training; each day included four trials with different starting points for each trial. The order of the start positions was not repeated throughout the testing days. A trial started by placing a rat gently into the water tail-end first, facing the outer edge of the pool. The daily average time spent to reach the platform was calculated, and each trial was terminated when the animal reached the platform and remained above it for 15 s before it would be taken out. On the other hand, if the animal did not reach the platform within 90 s, the animal was gently guided toward the platform and was allowed to sit on it for 15 s. Then, the animals were transferred to a dry holding cage after being dried off with a towel. On the 6^th^ day, a thirty-second probe trial was performed (24 h after the last training session) during which the platform was removed, and rats were placed in a new start position. The time spent within the target quadrant (which formerly contained the platform during training sessions) was presented as a percentage of the total trial length. A camera mounted on the ceiling was placed directly above the pool to record the swim path of each rat [38].

#### Attentional Set-Shifting Task (ASST)

The used protocol was modeled after the previously described protocol [40]. The testing apparatus is a rectangular plexiglass/aluminum arena (45 × 70 × 25 cm) containing a removable divider separating 1/3 of the arena’s length from the rest to form a start box. A panel divided the opposite third of the arena into two sections with a pot placed in each section.

Eight days before testing, rats were subjected to a restricted diet (14–15 g per day). Each rat was allowed daily 20 min of free exploration of the testing apparatus in the absence of any testing stimuli. A small piece of cereal was provided in the rat’s home cage to familiarize it with the odor and taste of the food reward.

After 6 days of testing apparatus habituation, rats were trained for 2 days to dig in pots to get the food reward with the pot filled with cage bedding. The presence or absence of food reward in a pot was indicated by either olfactory stimuli (scent of the digging medium) or tactile stimuli (type of the digging medium). Animals were trained to dig for food until the criterion of 6 consecutive trials with the correct choice of digging pot was reached.

On the ninth day (experimental day), testing started with simple discrimination (SD) with the pots differing only in one dimension. After completion of the required criterion, the rats were tested for compound discrimination (CD) using the same pots of the SD with the same rewarded dimension but a second, irrelevant dimension was introduced. After reaching the required criterion, the reinforcement rules were reversed, and the rat had to recognize that the previously correct stimulus within the rewarded dimension was now incorrect; this stage was termed reversal-1 (REV1). After rats achieved the criterion for REV1, they were tested for the intra-dimensional shift (IDS). During the IDS, the rats were presented with a novel set of stimuli but were required to attend to the same perceptual dimension that had been reinforced during the SD, CD, and REV1. The rats then received a reversal of IDS (REV2). Then, rats were tested on the extra-dimensional shift (EDS) during which, novel stimuli were presented again; now, the rat had to stop tracking the previously relevant dimension and shift attention to the previously irrelevant dimension that was now rewarding. After the criterion was reached for the EDS, a reversal of EDS (REV3) was done.

All dimensions and pairs of exemplars were equally represented within groups. Every rat was allowed 3 min per trial to make a choice. Animals had free access to water in the waiting area to encourage them to eat and to prevent thirst during the test. The number of trials made by each rat to reach the criterion for each stage was recorded.

### Biochemical and Molecular Studies

#### Sacrification and Sample Collection

Twenty-four hours following the last drug dose, rats were anesthetized using urethane 1.2 g/ kg, i.p. [38]. For biochemical assays (10 rats/group), trans-cardiac perfusion through the left ventricle with 40 ml PBS (pH 7.4) was done. After that, rats were decapitated, the brain was dissected on an ice-cold plate. the hippocampus was rapidly collected and immediately frozen (-80 ℃). For histopathological studies (8 rats/group), trans-cardiac perfusion through the left ventricle with 40 ml 10% neutral buffered formalin was done. After that, rats were decapitated and the whole brain was dissected, post-fixed in 10% neutral buffered formalin, and processed to form paraffin blocks [41].

#### Western-Blot Analysis for Detection of Total and Phosphorylated cAMP Response Element-binding Protein (Total CREB and p-CREB)

Each brain sample containing both right and left hippocampi was dissected with a surgical sterile blade in a sterile plate. The resulting homogenate was then divided into two parts (approximately equal to each other); the first was used to perform ELISA and western-blot analysis while the second part was utilized for HPLC and RT-qPCR analysis.

Protein concentration in each sample was determined by the Bradford technique [42] using Bradford protein assay kit (cat# SK3041; Bio Basic Inc., Markham, ON, Canada). Then, a 20 μg protein concentration of each sample was loaded with an equal volume of 2 × Laemmli sample buffer containing 4% SDS, 10% 2-mercaptoethanol, 20% glycerol, 0.004% bromophenol blue, and 0.125 M Tris HCl at pH 6.8. Polyacrylamide gels were performed using TGX Stain-Free™ FastCast™ Acrylamide Kit (cat# 161–0181; Bio-Rad Lab, Hercules, CA, USA). The gel was assembled in a transfer sandwich as follows from below to above (filter paper, PVDF membrane, gel, and filter paper). The sandwich was placed in the transfer tank with 1 × transfer buffer composed of 25 mM Tris and 190 mM glycine and 20% methanol. The blot was then run for 7 min at 25 V to allow protein bands to transfer from gel to membrane using the Bio-Rad Trans-Blot Turbo transfer system. The membrane was blocked in tris-buffered saline with Tween 20 (TBST) buffer and 3% bovine serum albumin (BSA) at room temperature for 1 h. The components of the blocking buffer were as follows; 20 mM Tris pH 7.5, 150 mM NaCl, 0.1% Tween 20, and 3% bovine serum albumin (BSA).

The used primary antibodies were CREB (cat# sc-377154; Santa Cruz Biotechnology, Santa Cruz, CA, USA) and Phospho-CREB (Ser133) (cat# 9198; Cell Signaling Technology, Danvers, MA, USA). Incubation was done overnight with each primary antibody solution, against the blotted target protein, at 4 °C. The blot was rinsed 3–5 times for 5 min with TBST. Incubation was done in Goat anti-Rabbit IgG Secondary Antibody [HRP] (cat# NB7160; Novus Biologicals, Centennial, CO, USA) solution against the blotted target protein for 1 h at room temperature. The blot was rinsed 3–5 times for 5 min with TBST.

The chemiluminescent substrate “Clarity™ Western ECL substrate” (cat# 1705060; Bio-Rad Lab) was applied to the blot according to the manufacturer’s instructions. The chemiluminescent signals were captured using a CCD camera-based imager. Image analysis software was used to read the band intensity of the target proteins against the control sample beta-actin (housekeeping protein) by protein normalization on the Bio-Rad ChemiDoc™ MP imager.

#### Estimation of Gene Expression of *Glt-1* Using Real-Time Quantitative Polymerase Chain Reaction (RT-qPCR) Analysis

Total RNA was extracted from homogenized samples using Direct-zol RNA Miniprep Plus (cat# R2072; Zymo Research, Irvine, CA, USA) according to the manufacturer’s instructions. SuperScript^TM^ IV One-Step RT-PCR kit (Cat# 12594100, Thermo Fisher Scientific, Waltham, MA, USA) was utilized for reverse transcription of extracted RNA followed by PCR. 48-well plate StepOne instrument (Applied Biosystem, Waltham, MA, USA) was used in a thermal profile as follows: 10 min at 45 ºC for reverse transcription, 2 min at 98 ºC for RT inactivation and initial denaturation by 40 cycles of 10 s at 98 ºC, 10 s at 55 ºC and 30 s at 72 ºC for the amplification step. After the RT-PCR run, the data was expressed in Cycle threshold (Ct) for the target genes and housekeeping gene. For *Glt-1*, the forward primer sequence was 5′-TGACAAGCGTGTGACCAGATTCG-3′ and the reverse primer sequence was 5′-GCTCGCCAGAGTTGCTGTAAGG-3′. The glyceraldehyde-3-phosphate dehydrogenase (*Gapdh*) was used as the reference gene, the forward primer sequence was 5'-CCTCGTCTCATAGACAAGATGGT-3' and the reverse primer sequence was 5'- GGGTAGAGTCATACTGGAACATG-3'. The 2^− ΔΔCt^ method was conducted for the analysis of gene expression levels [43].

#### Estimation of Brain-Derived Neurotrophic Factor (BDNF) Using Enzyme-Linked Immunosorbent Assay (ELISA)

BDNF protein was measured in hippocampal tissue homogenates using rat BDNF ELISA kit (cat# SEA011Ra; Cloud-Clone Corp., Katy, Tx, USA) according to the manufacturer’s instructions. Absorbance was measured at 450 nm. The protein concentration of hippocampal homogenates was measured using the Bradford technique [40].

#### High-Performance Liquid Chromatography (HPLC) for Measuring Glutamate and γ-Aminobutyric Acid (GABA) Levels

HPLC was used to measure glutamate and GABA levels, as previously described by John et al. [44]. The hippocampi were homogenized in an ice-cold solution (8.8 mg of ascorbic acid and 122 mg of EDTA in 1000 ml of perchloric acid 0.1 mol/l). The homogenate was then centrifuged at 10,000 g for 10 min at 4 °C (Beckman GS 15R bench-top cooling centrifuge; Beckman Coulter, Brea, CA, USA). The supernatant obtained was subjected to HPLC analysis that was carried out using an Agilent 1260 series using Eclipse Plus C18 column (4.6 mm × 250 mm internal diameter, 5 μm) (Markham, ON, Canada). The mobile phase contained 50 mM phosphate buffer (KH2PO4/K2HPO4) containing 10% v/v methanol, pH 6.2 (solvent A), and methanol (solvent B) with a flow rate of 1.5 ml/min. All solvents are HPLC-grade (Sigma-Aldrich, St. Louis, MO, USA). The fluorescence detector was adjusted at 340/450 nm (Excitation/Emission). Glutamate (Glu) and GABA levels were calculated from the peak area of each compound and compared with their standard curves (Fig. 5E).

### Histopathological Studies

Rats’ brain tissue samples were fixed in 10% neutral buffered formalin for 72 h. Samples were processed in serial grades of ethanol, cleared in Xylene, and embedded into Paraplast tissue embedding media (Leica Biosystems, Wetzlar, Germany). 5 μm thick serial sagittal brain sections were cut by rotatory microtome and mounted on glass slides. Tissue sections were stained with Hematoxylin and Eosin (H&E) as a standard staining method for light microscopic examination, toluidine blue staining for determination of the mean number of intact neurons and the mean optical density of Nissl’s granules which are depleted in neurodegeneration, and Marsland, Glees and Erikson's silver impregnation technique for detection of neurofibrillary tangles (NFTs) which appear as dark brown argyrophilic aggregates. For immunohistochemical staining, 5 µm sections were cut and mounted on positively charged slides for the Avidin–Biotin Peroxidase technique using primary antibodies for Glial Fibrillary Acidic Protein (GFAP) (1:1000) (cat# AB5804; Sigma-Aldrich, St. Louis, MO, USA), cleaved caspase-3 (1:1500) (cat# GB11532, Wuhan Servicebio Biotechnology, Wuhan, China), BAX (1:100) (cat# 33–6600; Thermo Fisher Scientific, Fremont, CA, USA), and Bcl-2 (1:100) (cat# PA5-27094; Thermo Fisher Scientific, Fremont, CA, USA). After primary antibody incubation, slides-mounted tissue sections were incubated with biotinylated anti-rabbit secondary antibody (1:2000) (cat# BA-9200; Vector Lab., Burlingame, CA, USA) for 30 min. Finally, the slides were counterstained with hematoxylin, dehydrated, cleared, and mounted.

Cell quantification was conducted by an experienced histologist who was blinded to the treatments. At least 3 different sections were analyzed per each sample and morphometric measurements were obtained from 6 different non-overlapping fields for each slide to measure the mean intact neurons count, mean optical density of Nissl’s granules as well as the mean area percentage of GFAP, caspase-3, BAX, and Bcl-2 immune reaction. Morphometric analysis was performed using the Leica QWin V.3 image analysis software (Leica Microsystems, Wetzlar, Germany). Images were captured onto the screen from sections under the light microscope (Olympus BX-40, Olympus Optical Co., Tokyo, Japan).

### Statistical Analysis

All values were expressed as mean ± S.E.M. Statistical analysis was carried out using the GraphPad prism software program, version 7.0 (2016) Inc., CA, USA. Data was tested for normality using D'Agostino and Pearson omnibus normality test. The statistical difference among groups was determined using ordinary one-way ANOVA followed by Tukey’s multiple comparisons test for comparison between more than two groups. Repeated-measures ANOVA was utilized for the analysis of body weight and time to reach the platform in MWM. *P* values < 0.05 were considered statistically significant.

## Results

### Body Weight

**Fig. 2:**
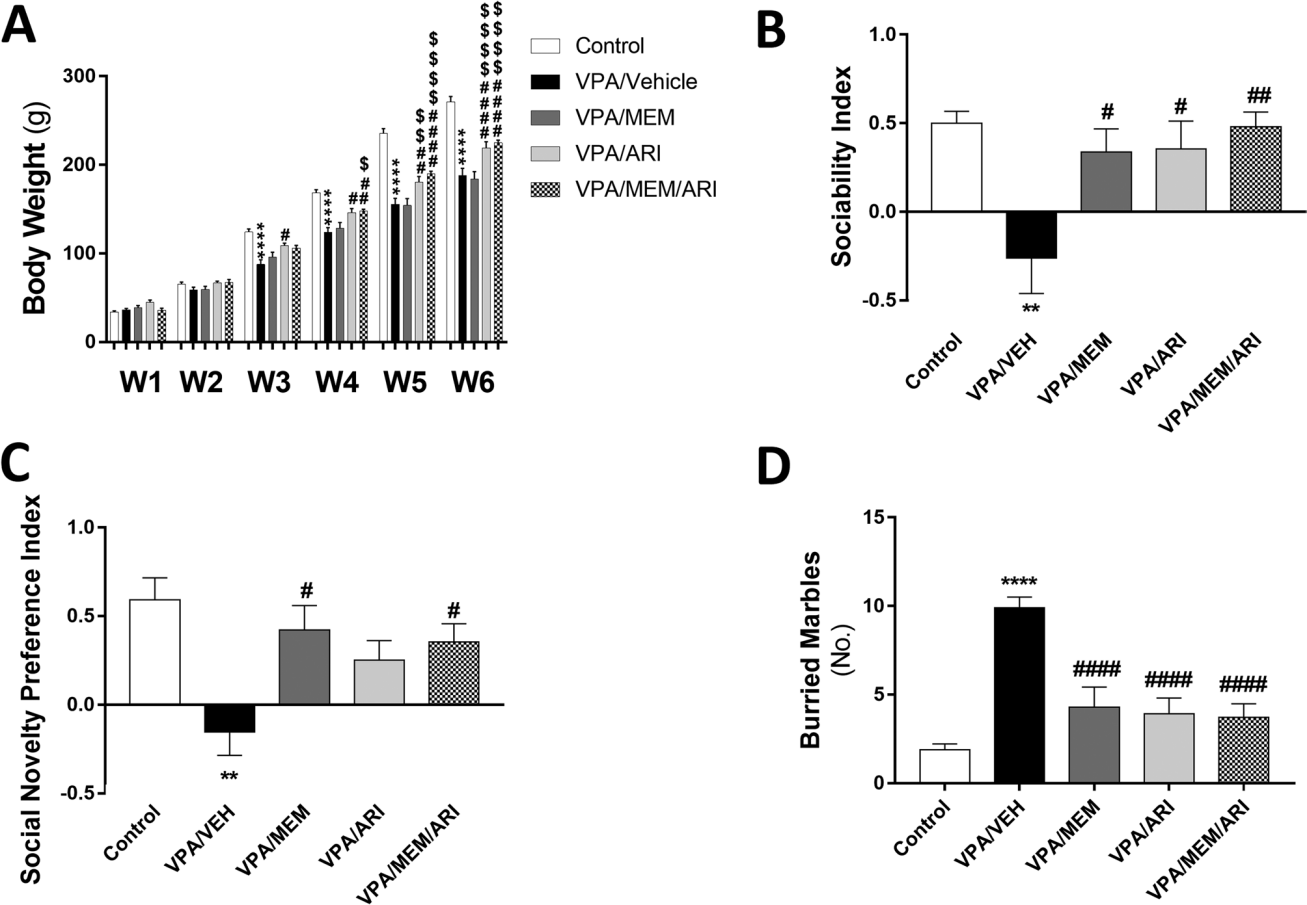
Effects of MEM and ARI on **A** body weight, **B** sociability index, **C** social novelty preference index, and **D** number of buried marbles in prenatal VPA rat model of autism. Data are presented as mean ± S.E.M (n = 10). ^**^P < 0.01; ^****^P < 0.0001 vs. Control group; ^#^*P* < 0.05; ^##^*P* < 0.01; ^####^*P* < 0.0001 vs. VPA/VEH group; ^$^*P* < 0.05; ^$$^*P* < 0.01; ^$$$$^*P* < 0.0001 vs. VPA/MEM group. Treatments were compared by one-way ANOVA followed by Tukey's post-hoc test. For body weight, treatments were compared by repeated-measures ANOVA followed by Tukey's post-hoc test

As shown in Fig. 2A, repeated-measures ANOVA revealed a significant main effect of time (F _(5, 225)_ = 3453, *P* < 0.0001), treatment (F _(4, 45)_ = 16.48, *P* < 0.0001) and a significant interaction between the time factor and the treatment factor (F _(20, 225)_ = 33.87, *P* < 0.0001). Prenatal VPA exposure induced a significant decrease in body weight at the end of the 3^rd^, 4^th^, 5^th^, and 6^th^ weeks (29.33%, 26.54%, 34.06%, and 30.65% respectively) compared to the control group. VPA/ARI group exhibited a significant increase in body weight at the end of the 3^rd^, 4^th^, 5^th^, and 6^th^ weeks compared to the VPA/VEH group (24.06%, 17.95%, 16.03%, and 16.54% respectively) and at the end of the 5^th^ and 6^th^ weeks compared to the VPA/MEM group (16.94% and 19.14% respectively). VPA/MEM/ARI group exhibited a significant increase in body weight at the end of the 4^th^, 5^th^, and 6^th^ weeks compared to the VPA/VEH group (19.24%, 22.41%, and 19.57% respectively) and compared to the VPA/MEM group (14.96%, 23.36%, and 22.24% respectively).

### Behavioral Changes

#### Three-Chamber Sociability Test

One-way ANOVA revealed a significant main effect of treatments (F _(4, 45)_ = 5.115, *P* < 0.01; F _(4, 45)_ = 5.132, *P* < 0.01 for sociability index and social novelty preference index respectively). VPA/VEH group exhibited a significant decrease in sociability and social novelty preference indices (152.5% and 126.21% respectively) compared to the control group. Treatment with MEM and ARI monotherapy and in combination induced a significant increase in sociability index (228.76%, 235.62%, and 282.86% respectively) compared to the VPA/VEH group. As regards, the social novelty preference index, only VPA/MEM and VPA/MEM/ARI groups exhibited a significant increase (372.26% and 328.39% respectively) compared to VPA/VEH group (Fig. 2B, C).

#### Marble Burying Test

As shown in Fig. 2D, one-way ANOVA revealed a significant main effect of treatments (F _(4, 45)_ = 14.48, *P* < 0.0001). VPA/VEH group exhibited a significant increase in the number of buried marbles (421.05%) compared to the control group. VPA/MEM, VPA/ARI, and VPA/MEM/ARI groups showed a significant decrease in the number of buried marbles (56.57%, 60.61%, and 62.63% respectively) compared to the VPA/VEH group.

#### Open Field Test

**Fig. 3:**
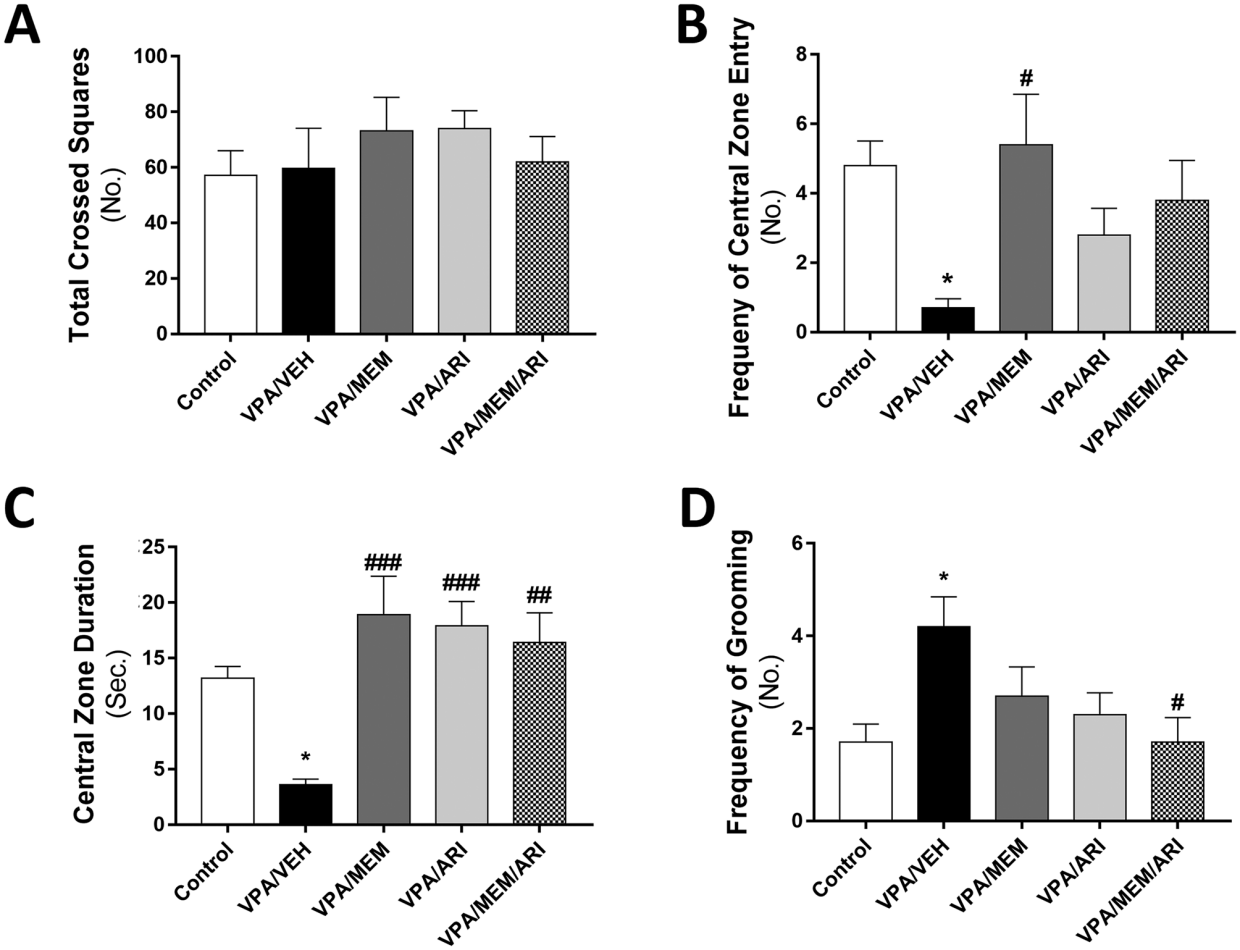
Effects of MEM and ARI on open field behavioral changes in prenatal VPA rat model of autism. Data are presented as mean ± S.E.M (n = 10). ^*^P < 0.05 vs. Control group; ^#^*P* < 0.05; ^##^*P* < 0.01; ^###^*P* < 0.001 vs. VPA/VEH group. Treatments were compared by one-way ANOVA followed by Tukey's post-hoc test

As depicted in Fig. 3A–D, one-way ANOVA revealed a significant main effect of treatments (F _(4, 45)_ = 3.717, *P* < 0.05; F _(4, 45)_ = 7.482, *P* < 0.001; F _(4, 45)_ = 3.566, *P* < 0.05 for frequency of entry to the central zone, central zone duration and frequency of grooming respectively). VPA/VEH group exhibited a significant decrease in the frequency of entry to the central zone and central zone duration (85.42% and 72.73% respectively) with a significant increase in the frequency of grooming (147.06%) compared to the control group. VPA/MEM group exhibited a significant increase in the frequency of entry to the central zone and central zone duration (671.43% and 425% respectively) compared to the VPA/VEH group. VPA/ARI group showed a significant increase in central zone duration (397.22%) compared to VPA/VEH group. VPA/MEM/ARI group exhibited a significant increase in central zone duration (355.56%) alongside a decrease in the frequency of grooming (59.52%) compared to the VPA/VEH group.

#### Morris Water Maze

**Fig. 4:**
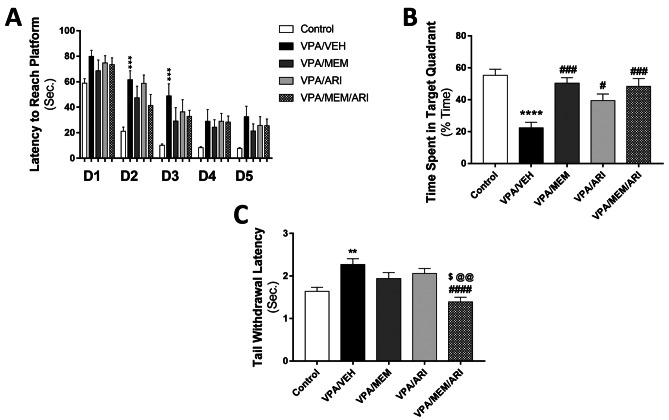
Effects of MEM and ARI on **A** latency to reach MWM hidden platform, **B** % time spent in the target quadrant in MWM, and **C** TNT tail withdrawal latency in prenatal VPA rat model of autism. Data are presented as mean ± S.E.M (n = 10). ^**^P < 0.01; ^***^P < 0.001; ^****^P < 0.0001 vs. Control group; ^#^*P* < 0.05; ^###^*P* < 0.001; ^####^*P* < 0.0001 vs. VPA/VEH group; ^$^*P* < 0.05 vs. VPA/MEM group; ^@@^*P* < 0.01 vs. VPA/ARI group. Treatments were compared by one-way ANOVA followed by Tukey's post-hoc test. For the latency to reach MWM hidden platform, treatments were compared by repeated-measures ANOVA followed by Tukey's post-hoc test

As regards the latency to reach the platform during the spatial acquisition learning task, repeated-measures ANOVA revealed a significant main effect of time (F _(4,180)_ = 51.06, *P* < 0.0001) and treatment (F _(4,45)_ = 9.89, *P* < 0.0001). VPA/VEH group exhibited a significant increase in the latency to reach the platform on the 2^nd^ and 3^rd^ days compared to the control group (Fig. 4A).

As regards the probe trial, one-way ANOVA revealed a significant main effect of treatments (F _(4, 45) =_ 10.51, *P* < 0.001). VPA/VEH group showed a significant decrease in % time spent in the target quadrant (59.62%) compared to the control group. VPA/MEM, VPA/ARI and VPA/MEM/ARI groups exhibited a significant increase in % time spent in the target quadrant (125.26%, 76.13%, and 116.44% respectively) compared to VPA/VEH group (Fig. 4B).

#### Attentional Set-Shifting Task

As shown in Table 1, one-way ANOVA revealed a significant main effect of treatments (F _(4, 35)_ = 7.868, *P* < 0.001; F _(4, 35)_ = 12.66, *P* < 0.0001; F _(4, 35)_ = 21.78, *P* < 0.0001; F _(4, 35)_ = 13.86, *P* < 0.0001; F _(4, 35)_ = 19.25, *P* < 0.0001 for CD, REV1, REV2, EDS, and REV3 respectively). VPA/VEH group exhibited a significant increase in the number of trials to reach the criterion in the domains of CD (64.60%), REV1 (36.09%), REV2 (118.4%), EDS (23.67%), and REV3 (28.97%) compared to the control group. VPA/MEM and VPA/MEM/ARI groups exhibited a significant decrease in the number of trials to reach the criterion in the domains of CD (39.27%), REV1 (22.21% and 20.51% respectively), REV2 (51.16% and 41.21% respectively), EDS (42.68% and 29.20% respectively), and REV3 (42.68% and 39.35% respectively) while VPA/ARI group showed a significant decrease only in CD (39.27%), REV2 (41.21%), EDS (25.88%) and REV3 stages (35.94%) compared to the VPA/VEH group.

**Table 1:** Effects of MEM and ARI on number of trials to reach criterion in the attentional set-shifting task in prenatal VPA rat model of autism

**Groups**	**SD**	**CD**	**REV1**	**IDS**	**REV2**	**EDS**	**REV3**
**Control**	6.38 ± 0.38	6.00	10.75 ± 0.49	8.38 ± 0.60	7.50 ± 0.63	9.00 ± 0.38	8.63 ± 0.57
**VPA/VEH**	6.25 ± 0.16	9.88 ± 1.38 ^***^	14.63 ± 0.46 ^****^	9.38 ± 0.99	16.38 ± 1.02 ^****^	11.13 ± 0.44 ^*^	11.13 ± 0.61 ^**^
**VPA/MEM**	6.38 ± 0.26	6.00 ^###^	11.38 ± 0.42 ^####^	7.00 ± 0.38	8.00 ± 0.82 ^####^	6.38 ± 0.32 ^####^	6.38 ± 0.26 ^####^
**VPA/ARI**	6.50 ± 0.27	6.00 ^###^	13.00 ± 0.42	9.50 ± 0.73	9.63 ± 0.57 ^####^	8.25 ± 0.49 ^##^	7.13 ± 0.35 ^####^
**VPA/MEM/ARI**	6.13 ± 0.13	6.00 ^###^	11.63 ± 0.38 ^###^	8.38 ± 0.68	9.63 ± 0.71 ^####^	7.88 ± 0.64 ^###^	6.75 ± 0.31 ^####^

Data are mean ± S.E.M (n = 8)

*SD* Simple Discrimination, *CD* Compound Discrimination, *REV1* Reversal 1, *IDS* Intra-dimensional Shift, *REV2* Reversal 2, *EDS* Extra-dimensional Shift, *REV3* Reversal 3

^*^P < 0.05; ^**^P < 0.01; ^***^P < 0.001; ^****^P < 0.0001 vs. Control group; ^##^*P* < 0.01; ^###^*P* < 0.001; ^####^*P* < 0.0001 vs. VPA/VEH group by One-Way ANOVA followed by Tukey's post-hoc test

#### Tail-Immersion Nociceptive Test

One-way ANOVA revealed a significant main effect of treatments (F _(4, 45)_ = 8.244, *P* < 0.0001). VPA/VEH group exhibited a significant increase in tail withdrawal latency (38.41%) compared to the control group. VPA/MEM/ARI group showed a significant decrease in tail withdrawal latency compared to the VPA/VEH, VPA/MEM, and VPA/ARI groups (38.77%, 28.35%, and 32.52%) (Fig. 4C).

### Biochemical Measurements

#### GABA/Glutamate Balance

**Fig. 5:**
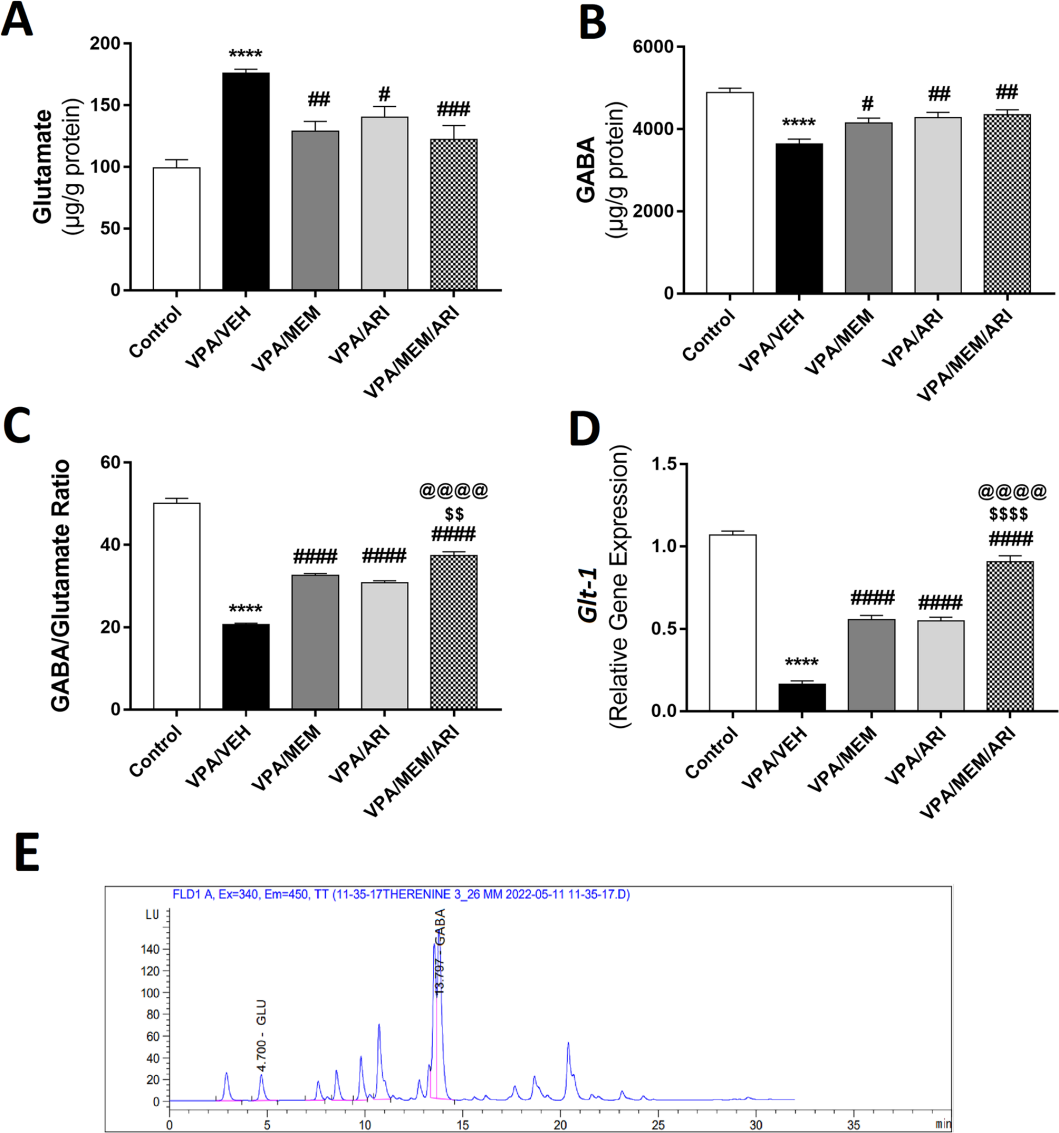
Effects of MEM and ARI on **A** Glutamate level, **B** GABA level, **C** GABA/glutamate ratio, and **D** *Glt-1* relative gene expression in prenatal VPA rat model of autism. Data are presented as mean ± S.E.M (n = 6). ^****^P < 0.0001 vs. Control group; ^#^*P* < 0.05; ^##^*P* < 0.01; ^###^*P* < 0.001; ^####^*P* < 0.0001 vs. VPA/VEH group; ^$$^*P* < 0.01; ^$$$$^*P* < 0.0001 vs. VPA/MEM group; ^@@@@^*P* < 0.0001 vs. VPA/ARI group. Treatments were compared by one-way ANOVA followed by Tukey's post-hoc test. **E** Example of HPLC chromatogram showing Glutamate (Glu) and GABA peaks

As shown in Fig. 5A–C, one-way ANOVA revealed a significant main effect of treatments (F _(4,25)_ = 12.21, *P* < 0.0001; F _(4,25)_ = 13.86, *P* < 0.0001; F _(4,25)_ = 204.2, *P* < 0.0001 for glutamate, GABA, and GABA/glutamate ratio respectively). VPA/VEH group exhibited a significant increase in glutamate and a decrease in GABA, with a decrease in GABA/glutamate ratio (76.97%, 25.58%, and 58.45% respectively) compared to the control group. VPA/MEM and VPA/ARI groups showed a significant decrease in glutamate (26.76% and 20.23% respectively) alongside an increase in GABA (14.19% and 17.62% respectively) and GABA/glutamate ratio (56.46% and 47.37% respectively) compared to the VPA/VEH group. MEM and ARI combined administration revealed a significant decrease in glutamate (30.57%) and an increase in GABA and GABA/glutamate ratio (19.61% and 79.43% respectively) compared to the VPA/VEH group with a significant increase in GABA/glutamate ratio compared to the VPA/MEM (14.68%) and the VPA/ARI groups (21.75%).

#### *Glt-1* Relative Gene Expression

As depicted in Fig. 5D, one-way ANOVA revealed a significant main effect of treatments (F _(4, 25)_ = 182.8, *P* < 0.0001). VPA/VEH group exhibited a significant decrease in *Glt-1* relative gene expression compared to the control group (84.64%). MEM and ARI monotherapy induced a significant increase in *Glt-1* relative gene expression (239.19% and 233.91% respectively) compared to the VPA/VEH group. Whereas VPA/MEM/ARI group showed an increased *Glt-1* gene expression compared to VPA/VEH, VPA/MEM, and VPA/ARI groups (452.22%, 62.81%, and 65.38% respectively).

#### Total and Phosphorylated CREB Protein

**Fig. 6:**
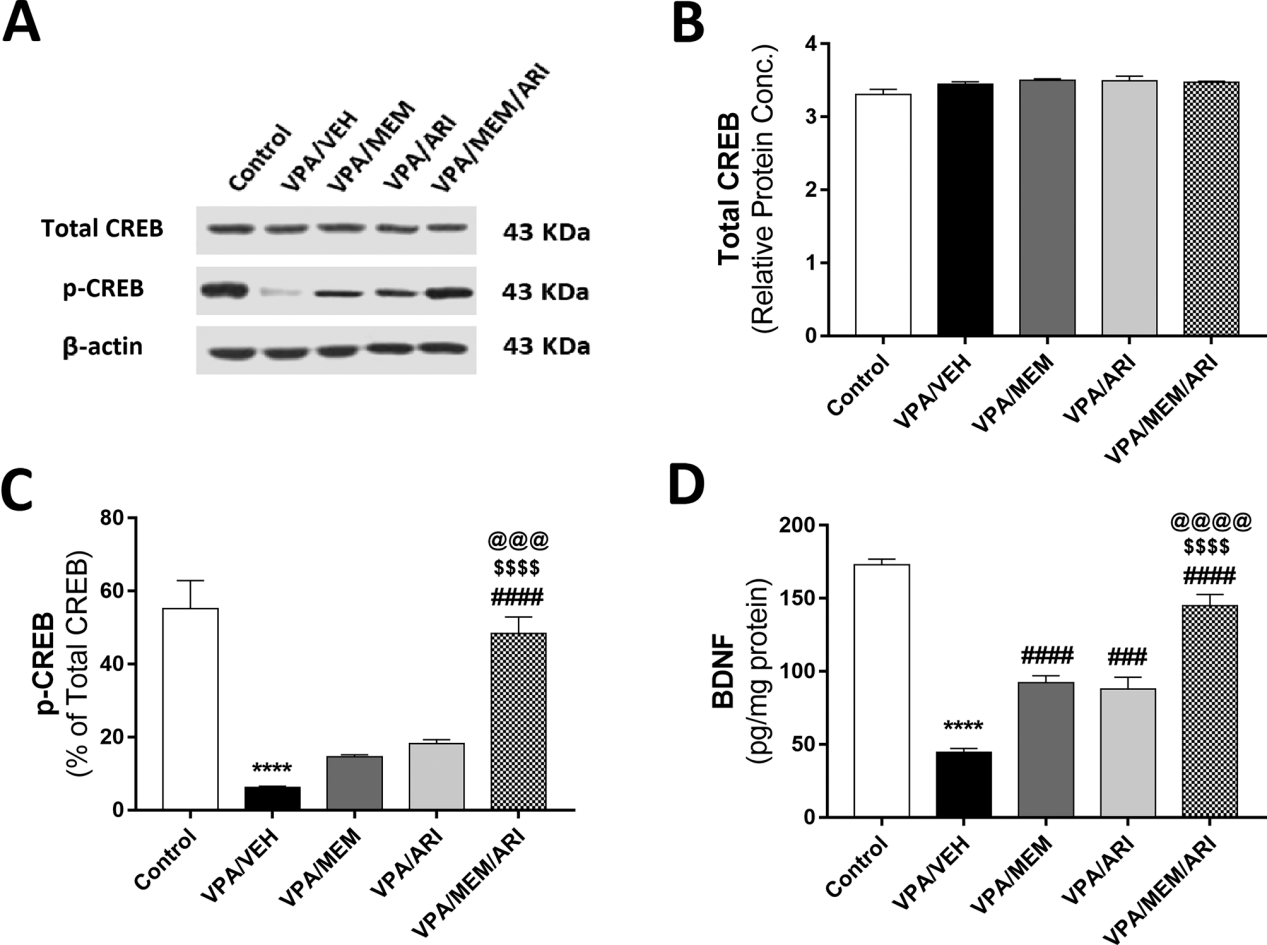
Effects of MEM and ARI on protein levels of total, p-CREB, and BDNF in prenatal VPA rat model of autism. **A** A representative image of quantitative protein levels of total and p-CREB normalized to β-actin, **B** Total CREB, **C** p-CREB, and **D** BDNF. Data are presented as mean ± S.E.M (n = 6). ^****^P < 0.0001 vs. Control group; ^###^*P* < 0.001; ^####^*P* < 0.0001 vs. VPA/VEH group; ^$$$$^*P* < 0.0001 vs. VPA/MEM group; ^@@@^*P* < 0.001; ^@@@@^*P* < 0.0001 vs. VPA/ARI group. Treatments were compared by one-way ANOVA followed by Tukey's post-hoc test

As shown in Fig. 6A–C, one-way ANOVA revealed a significant main effect of treatments (F _(4, 25)_ = 28.82, *P* < 0.0001 for p-CREB). VPA/VEH group exhibited a decline in p-CREB (88.73%) compared to the control group. Only VPA/MEM/ARI group showed a significant increase in the p-CREB compared to the VPA/VEH (678.6%), VPA/MEM (230.53%), and VPA/ARI (166.61%) groups.

#### BDNF Protein

One-way ANOVA revealed a significant main effect of treatments (F _(4, 25)_ = 73.43, *P* < 0.0001). VPA/VEH group exhibited a significant decrease in BDNF protein (74.22%) compared to the control group. However, MEM and ARI monotherapies were associated with a significant increase in BDNF protein (107.09% and 97.15% respectively) compared to the VPA/VEH group. Interestingly, MEM and ARI combined administration showed a significant increase in BDNF compared to the VPA/VEH, VPA/MEM, and VPA/ARI groups (225.56%, 57.21%, and 65.31% respectively) (Fig. 6D).

### Histological Studies

**Fig. 7:**
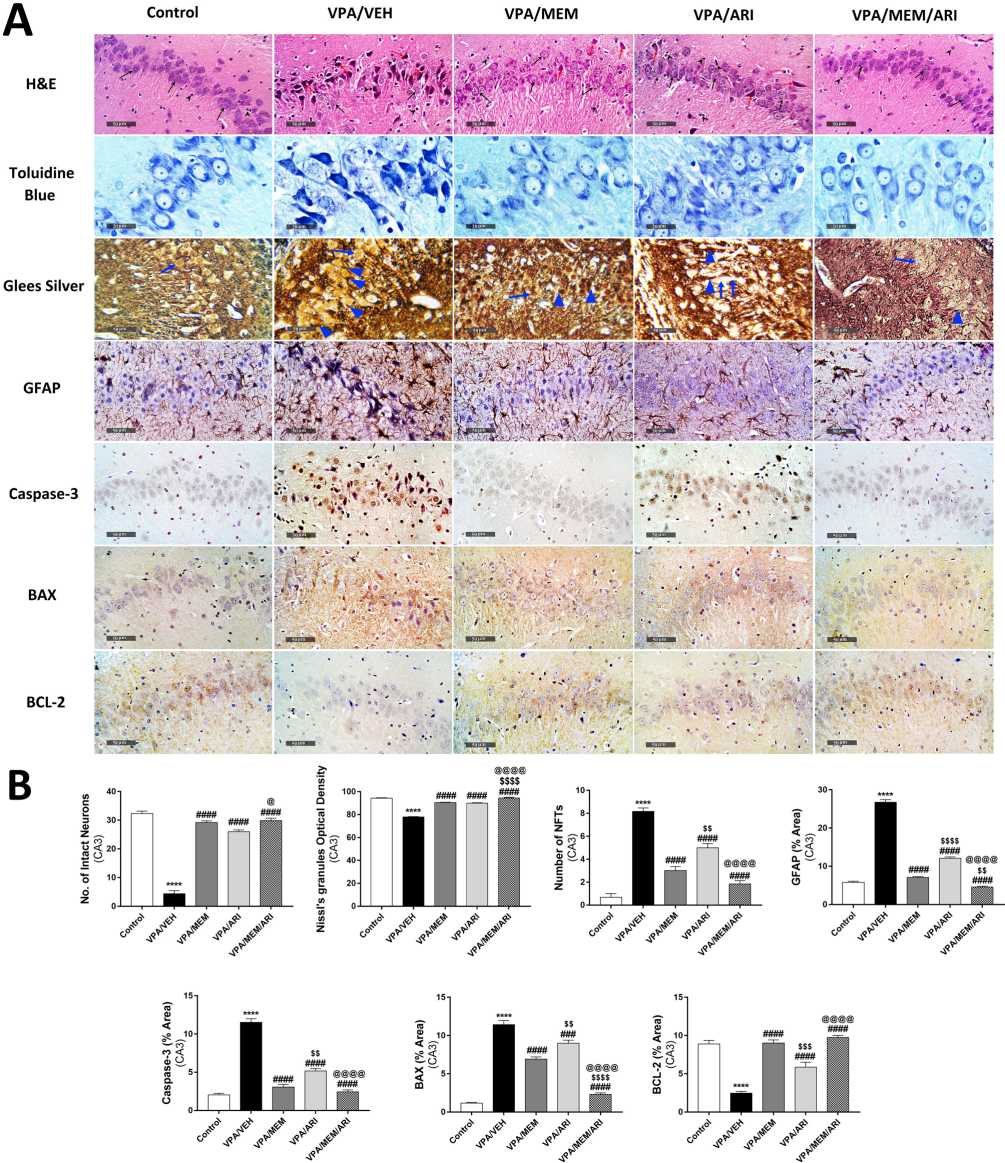
**A** Photomicrographs representing H&E, toluidine blue, Marsland, Glees and Erikson's Silver, GFAP, caspase-3, BAX, and Bcl-2-stained sections of CA3 hippocampal area from the study groups. The control group showed many intact well-organized neurons with distinct nuclear and subcellular details (black arrow) and minimal reactive glial cell infiltrates (black arrowhead) and normal neuronal argyrophilia (blue arrow) alongside an increased optical density of Nissl’s granules, a high immunoreactivity of Bcl-2, and a low immunoreactivity of GFAP, caspase-3, and BAX. VPA/VEH group showed abundant degenerated hypereosinophilic neurons without distinct subcellular details (red arrow) alternated with few dispersed intact cells (black arrow) with marked higher reactive glial cell infiltrates (black arrowhead) and several NFTs (blue arrowhead) alongside a low optical density of Nissl’s granules, low immunoreactivity of Bcl-2 and a high immunoreactivity of GFAP, caspase-3, and BAX. Treated groups showed improvement of all the histological changes that was obvious in the VPA/MEM/ARI group which exhibited many well-organized intact neurons and minimal reactive glial cell infiltrates with minimal NFTs besides a high optical density of Nissl’s granules, a high immunoreactivity of Bcl-2 and a low immunoreactivity of GFAP, caspase-3, and BAX. **B** Effects of MEM and ARI on the number of intact neurons, mean optical density of Nissl’s granules, number of NFTs, and area percentage of immunohistochemical expression of GFAP, caspase-3, BAX, and Bcl-2 in CA3 hippocampal area in prenatal VPA rat model of autism. Data are presented as mean ± S.E.M. (n = 6). ^****^P < 0.0001 vs. Control group; ^###^*P* < 0.001; ^####^*P* < 0.0001 vs. VPA/VEH group; ^$$^*P* < 0.01; ^$$$$^*P* < 0.0001 vs. VPA/MEM group; ^@^*P* < 0.05; ^@@@@^*P* < 0.0001 vs. VPA/ARI group. Treatments were compared by one-way ANOVA followed by Tukey's post-hoc test

**Fig. 8:**
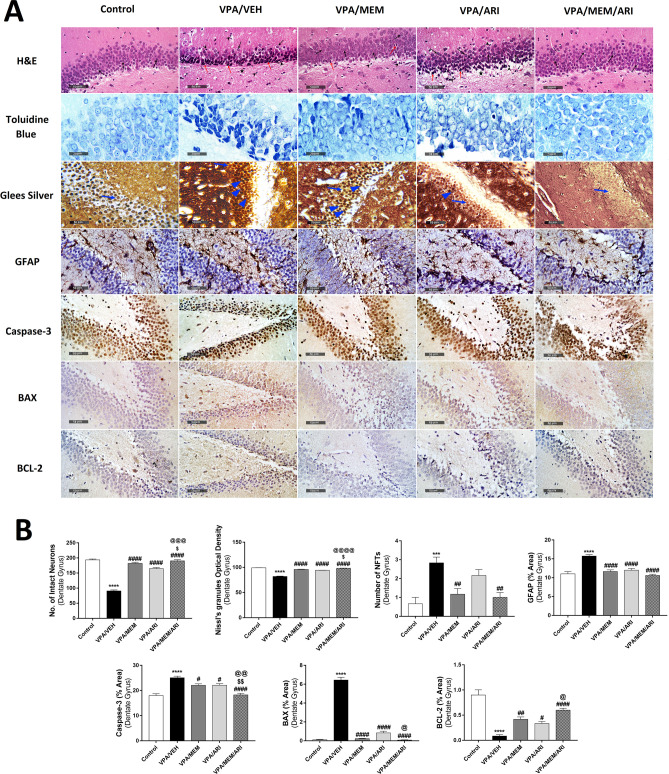
**A** Photomicrographs representing H&E, toluidine blue, Marsland, Glees and Erikson's Silver, GFAP, caspase-3, BAX, and Bcl-2-stained sections of dentate gyrus from the study groups. The control group exhibited granule cells with intact subcellular details (black arrow). VPA/VEH group showed marked degenerated pyknotic granule neurons (red arrow) with moderate edema and vacuolation of brain matrix (star) accompanied by mild higher reactive glial cell infiltrates (arrowhead) with multiple NFTs (blue arrowhead). Treated groups showed improvement of all the histological changes, especially in the VPA/MEM/ARI group which showed preserved morphological features with abundant intact neurons (black arrow) and minimal sporadic degenerated granule cells (red arrow). **B** Effects of MEM and ARI on the number of intact neurons, mean optical density of Nissl’s granules, number of NFTs, and area percentage of immunohistochemical expression of GFAP, caspase-3, BAX, and Bcl-2 in the dentate gyrus in prenatal VPA rat model of autism. Data are presented as mean ± S.E.M. (n = 6). ^***^P < 0.001; ^****^P < 0.0001 vs. Control group; ^#^*P* < 0.05; ^##^*P* < 0.01; ^####^*P* < 0.0001 vs. VPA/VEH group; ^$^*P* < 0.05; ^$$^*P* < 0.01 vs. VPA/MEM group; ^@^*P* < 0.05; ^@@^*P* < 0.01; ^@@@^*P* < 0.001; ^@@@@^*P* < 0.0001 vs. VPA/ARI group. Treatments were compared by one-way ANOVA followed by Tukey's post-hoc test

As shown in Figs. 7 and 8, the control group showed a normal morphological structure of the CA3 area and dentate gyrus with many apparent intact well-organized neurons with distinct nuclear and subcellular details (black arrow) and intact intercellular matrix with minimal reactive glial cell infiltrates (black arrowhead) and normal neuronal argyrophilia (blue arrow) alongside an increased optical density of Nissl’s granules, increased immunoreactivity of Bcl-2 and a low immunoreactivity of GFAP, caspase-3, and BAX. The VPA/VEH group showed abundant degenerated hypereosinophilic neurons without distinct subcellular details (red arrow) alternated with few dispersed apparent intact cells (black arrow). Moderate edema and vacuolation (star) and mild perineuronal edema were shown in the brain matrix with marked higher reactive glial cell infiltrates (black arrowhead) and several NFTs (blue arrowhead) alongside a low optical density of Nissl’s granules, a low immunoreactivity of Bcl-2, and a high immunoreactivity of GFAP, caspase-3, and BAX. VPA/MEM group exhibited occasional degenerated neurons (red arrow) and higher figures of intact, well-organized neurons (black arrow), mild reactive glial cell infiltrates (arrowhead) with a normal appearance of brain matrix. VPA/ARI group exhibited a persistent mild occasional neuronal damage (red arrow) alternated with intact neurons (black arrow) accompanied by moderate vacuolation and edema (star) as well as glial cell infiltrates (arrowhead). VPA/MEM/ARI group showed many well-organized intact neurons as well as minimal reactive glial cell infiltrates without abnormal morphological changes with minimal NFTs besides an increased optical density of Nissl’s granules and an increased immunoreactivity of Bcl-2 and a low immunoreactivity of GFAP, caspase-3, and BAX.

As regards the CA3 area, one-way ANOVA revealed a significant main effect of treatments (F _(4,25)_ = 187, *P* < 0.0001; F _(4,25)_ = 214.2, *P* < 0.0001; F _(4,25)_ = 76.79, *P* < 0.0001; F _(4,25)_ = 491.7, *P* < 0.0001; F _(4,25)_ = 137.4, *P* < 0.0001; F _(4,25)_ = 162, *P* < 0.0001; F _(4,25)_ = 49.9, *P* < 0.0001 for the number of intact neurons, Nissl’s granules mean optical density, the number of NFTs, and area percentage of GFAP, caspase-3, BAX, and Bcl-2 respectively). VPA/VEH group exhibited a significant decrease in the number of intact neurons, Nissl’s granules mean optical density, and Bcl-2 area percentage alongside an increase in the number of NFTs and GFAP, caspase-3, and BAX area percentage compared to the control group. VPA/MEM and VPA/ARI groups showed a significant improvement in these measurements compared to the VPA/VEH group. Notably, VPA/MEM/ARI exhibited a significant improvement in all parameters compared to the VPA/VEH group with a significant increase in Nissl’s granules mean optical density and a decrease in GFAP and BAX area percentage compared to the VPA/MEM group, and a significant increase in the number of intact neurons, Nissl’s granules mean optical density, and Bcl-2 area percentage alongside a decrease in the number of NFTs and GFAP, caspase-3 and BAX area percentage compared to the VPA/ARI group.

As regards the dentate gyrus, one-way ANOVA revealed a significant main effect of treatments (F _(4,25)_ = 130.2, *P* < 0.0001; F _(4,25)_ = 200.9, *P* < 0.0001; F _(4,25)_ = 8.825, *P* = 0.0001; F _(4,25)_ = 20.87, *P* < 0.0001; F _(4,25)_ = 21.51, *P* < 0.0001; F _(4,25)_ = 279.2, *P* < 0.0001; F _(4,25)_ = 27.41, *P* < 0.0001 for the number of intact neurons, Nissl’s granules mean optical density, the number of NFTs, and area percentage of GFAP, caspase-3, BAX, and Bcl-2 respectively). VPA/VEH group exhibited a significant decrease in the number of intact neurons, Nissl’s granules mean optical density, and Bcl-2 area percentage with an increase in the number of NFTs and GFAP, caspase-3, and BAX area percentage compared to the control group. VPA/MEM and VPA/ARI groups exhibited an improvement in most of these measurements compared to the VPA/VEH group. VPA/MEM/ARI exhibited a significant improvement in all parameters compared to the VPA/VEH group with a significant increase in the number of intact neurons and Nissl’s granules mean optical density and a decrease in caspase-3 area percentage compared to the VPA/MEM group, and a significant increase in the number of intact neurons, Nissl’s granules mean optical density, and Bcl-2 area percentage alongside a decrease in caspase-3 and BAX area percentage compared to the VPA/ARI group.

## Discussion

In this work, we investigated the effects of memantine/aripiprazole combined administration on autistic-like behaviors and cognitive functions in the prenatal valproate rat model of autism and explored their effects on CREB/BDNF signaling, astrocytic *Glt-1* expression, and glutamate balance which could represent key pathological events associated with ASD.

The results of the present study indicate the ability of prenatal VPA exposure during the critical phase of brain development to induce the core autistic features manifested through the decreased sociability and social novelty preference indices denoting deficits in social communication, in addition to the increased number of buried marbles and frequency of grooming reflecting the repetitive stereotyped behavioral patterns while the decreased central zone duration/entries in OFT could be a corollary to ASD-associated anxiety.

Cognitive deficits in the domains of spatial learning (denoted by the impaired performance in MWM spatial navigation task) and cognitive flexibility (denoted by defective performance in ASST) reflect ASD-associated cognitive dysfunction, a common ASD-associated problem [45]. Notably, the defective performance in ASST was more prominent in the reversal stages referring to an impairment in the ability to make transitions from the preferred behavioral patterns to new, more adaptive ones which contributes to the restrictive and repetitive behavior in autistic subjects [46]. Moreover, VPA-exposed rats exhibited a reduced body weight, a frequently encountered manifestation of prenatal VPA exposure [33, 34] as well as a decrease in pain sensitivity, a commonly associated ASD feature [34].

Astrocytic *GLT-1* is responsible for more than 90% of total glutamate uptake [9]. In astrocytes, glutamate is converted to glutamine (by glutamine synthetase) which is then shuttled to presynaptic terminals and is used as a precursor for the synthesis of GABA and glutamate [47, 48]. Glutamate and GABA are the major excitatory and inhibitory neurotransmitters, respectively, and the imbalance between these two neurotransmitters can affect brain development. Impaired glutamate metabolism can result in glutamate excitotoxicity which could be regarded as one of the core etiologies of many neurodegenerative conditions, including ASD. Excitotoxicity causes persistent uncontrolled neuronal excitation with subsequent neuronal damage [49, 50]. Thus, *GLT-1* could be very important for maintaining synaptic GABA/glutamate balance and preserving intraneural calcium homeostasis [51].

This was echoed through our results; prenatal VPA exposure was associated with a reduction in *Glt-1* gene expression with an increase in hippocampal glutamate and a decrease in GABA level and GABA/glutamate ratio accompanied by a reduction in the number of viable hippocampal neurons, reduced Nissl’s granules expression and increased apoptotic mediators, BAX and caspase-3 and reduction in the anti-apoptotic regulator, Bcl-2.

These results are consistent with previous reports where prenatal VPA exposure enhanced excitatory glutamatergic and impaired inhibitory GABAergic synaptic transmission with exaggerated hippocampal apoptosis [52–55]. VPA exposure would affect not only neurons but also astrocytes; astrocytic *GLT-1* levels were found to be lower in the hippocampus of prenatally VPA-exposed rats [11]. Interestingly, decreased astrocytic *GLT-1* expression was shown to be associated with repetitive-like behavior and accelerated cognitive decline, whereas *Glt-1* overexpression was shown to attenuate cognitive deficits in transgenic mice [12, 56, 57].

In the current study, the reduced p-CREB may explain the VPA-induced decrease in *Glt-1* expression and subsequent neurotoxic effects; previous reports have linked lower levels of CREB in autistic individuals to increased symptom severity [58]. CREB is a cellular transcription factor that plays crucial roles in dendrite formation and spine growth as well as neuronal plasticity and long-term memory formation [59]. CREB can be phosphorylated by different kinases such as protein kinase A (PKA) and protein kinase C (PKC). Following phosphorylation at Ser133 and recruitment of cofactors, such as CREB binding protein (CBP), CREB can bind to a CRE sequence in the promoter region of downstream genes such as BDNF and GLT-1 [14, 15].

In turn, BDNF could activate its receptor "TrkB" leading to the activation of ERK with phosphorylation and subsequent increased transcriptional activity of CREB [16]. Notably, BDNF plays a vital role in regulating the development of dendrites and promoting dendritic complexity, moreover, the disturbance of BDNF expression is found in many ASD subjects [60].

Interestingly, prenatal VPA exposure was associated with an increase in the number of NFTs (intraneuronal deposits of the hyperphosphorylated microtubule-associated protein “tau”). These neurotoxic aggregates could be deeply involved in disrupting microtubular function and synaptic signaling with neuronal damage which represents a critical pathophysiological aspect of cognitive dysfunctions in ASD subjects [21, 34, 61].

In the current study, chronic combined treatment with memantine and aripiprazole alleviated VPA-induced cellular and behavioral deficits as evidenced by increased *Glt-1* gene expression, increased CREB and BDNF levels, improved GABA/glutamate balance, suppressed neuronal apoptosis, and decreased NFTs deposition. These were reflected on the behavioral level via the improvement of autistic-like behaviors and cognitive deficits induced by prenatal VPA exposure.

Memantine is a non-competitive NMDA receptor antagonist which has gained attention over the past years in the treatment of an array of diseases characterized by aberrant glutamatergic transmission such as Alzheimer's disease, Parkinson's disease, glaucoma, epilepsy, and neuropathic pain [25, 62–65]. Memantine can effectively block NMDA receptors in the presence of persistent pathological receptor over-stimulation but allows the transient physiological transmission necessary for maintaining memory and learning [25]. Moreover, memantine was shown to inhibit presynaptic glutamate release through the inhibition of voltage-dependent Ca^2+^ entry which was thought to be independent of NMDA receptors [66]. Hence, memantine can inhibit glutamate-mediated excitotoxicity while preserving the normal beneficial effects exhibited by NMDA receptors [67].

Memantine has been shown to improve receptive and expressive language, verbal recognition memory, social interaction, irritability, anxiety, stereotypic behavior, and hyperactivity in children with ASD [29–32, 68]. Previous studies report that memantine could reverse VPA-induced repetitive behaviors, anxiety, reduced social interaction, and exploratory activity deficits with amelioration of brain oxidative and nitrosative stress in prenatally VPA-exposed autistic rats and mice [26, 27].

On the other side, aripiprazole is an atypical antipsychotic with partial agonistic activity at the dopamine D_2_ and 5-HT_1A_ receptors approved by the FDA for the management of ASD-associated behavioral dysregulation in children and adolescents [22, 24]. Aripiprazole has been shown to improve cognitive abilities in autistic children with mild to moderate cognitive impairment [23]. Chronic aripiprazole treatment alleviated social interaction and recognition memory impairments and reversed the reduction in dendritic spine density in the prefrontal cortex and hippocampus of prenatally VPA-exposed mice [69]. Moreover, aripiprazole dose-dependently increased gene expression of *GLT-1* in rats’ hippocampus after 4 weeks of treatment [70].

Memantine/aripiprazole-associated increased p-CREB/BDNF levels reported in this study is consistent with other earlier reports [24, 28, 71, 72). Indeed, the enhanced p-CREB/BDNF expression could represent the basis for the enhanced *Glt-1* expression and the subsequent restoration of GABA/glutamate balance along with the improved neuronal survival shown in this work.

In conclusion, this study draws attention to the role of combination therapy with memantine and aripiprazole in the alleviation of prenatal VPA-induced autistic-like and cognitive deficits which could be mediated via blocking NMDA receptor-mediated excitotoxicity and enhancing CREB/BDNF signaling with increased expression of astrocytic *Glt-1* and subsequent restoration of GABA/glutamate balance and rescue of hippocampal neuronal survival.

## Supplementary Information

Below is the link to the electronic supplementary material.

Supplementary file1 (PDF 560 KB)

Supplementary file2 (PDF 526 KB)

Supplementary file3 (PDF 551 KB)

Supplementary file4 (PDF 508 KB)

Supplementary file5 (PDF 543 KB)

Supplementary file6 (PDF 568 KB)

Supplementary file7 (PDF 517 KB)

Supplementary file8 (PDF 534 KB)

## Data Availability

All data generated or analysed during this study are included in this published article.
